# A novel tapered guide extension catheter facilitated successful completion of complex percutaneous coronary intervention

**DOI:** 10.1186/s40001-023-01067-w

**Published:** 2023-02-25

**Authors:** Jianquan Liao, Runda Wu, Yuanji Ma, Meng Zhang, Yaolin Chen, Kang Yao, Junbo Ge

**Affiliations:** 1 Department of Cardiology, Zhongshan Hospital, Shanghai Institute of Cardiovascular Diseases, Fudan University, 180 Fenglin Road, Shanghai, 200032 China; 2 National Clinical Research Center for Interventional Medicine, Shanghai, China; 3 Department of Cardiology, Zhongshan Hospital, Fudan University (Xiamen Branch), Xiamen, China

**Keywords:** Guide extension catheter, Percutaneous coronary intervention, Backup support

## Abstract

**Background:**

Guide extension catheters (GEC) are widely applied to cope with insufficient backup support in complex percutaneous coronary intervention (PCI). In the study, we aim to evaluate the feasibility and safety with a novel 5-4F tapered GEC used in complex lesion.

**Methods:**

The single-center retrospective study enrolled a total of 615 patients, in whom the 5F or 5-4F Expressman GEC was used to facilitate PCI procedure. Demographic and procedural data were collected.

**Results:**

5F GEC was used in 295 patients and 5-4F tapered GEC in 320 patients. The average age was 63.6 ± 11.0 years and 81.6% of the patients were male. Severe calcification and chronic total occlusion (CTO) were the commonest indication for the GEC use. The 5-4F tapered GEC was frequently used in active greeting technique (AGT) during CTO intervention procedure than 5F GEC (6.1% vs. 13.1%, *p* < 0.001). The average depth of intubation was 41.5 ± 19.6 mm for the 5-4F tapered GEC and 24.4 ± 15.1 mm for 5F GEC (*p* < 0.001). The rate of successful device delivery with 5-4F GEC was higher than 5F GEC (95.6% vs. 98.4%, *p* = 0.037). Pressure damping with 5F GEC occurred frequently than 5-4F GEC (7.4% vs. 2.5%, *p* < 0.05). Similarly, the incidence of intraoperative hypotension was higher in 5F GEC than 5-4F GEC (4.7% vs.1.9%, *p* < 0.05).

**Conclusions:**

The novel 5-4F tapered GEC was superior to the 5F GEC in facilitating successful completion of PCI in the majority of patients with complex lesions via transradial approach.

## Introduction

Sufficient backup support is crucial for guide catheter in percutaneous coronary intervention (PCI). It is a dilemma echoed by interventional cardiologists when stent delivery is difficult in complex coronary lesion. Many strategies are applied to cope with insufficient backup support, including active/passive guiding-catheter support, buddy wire technique, anchor balloon technique and the mother–child guide extension catheter (GEC) technique [1]. The use of GEC facilitated successful completion of complex percutaneous coronary intervention [2–5]. Several GEC are commercially available, including Guidezilla II (Boston Scientific, USA), GuideLiner V3 (Teleflex, USA), Telescope GEC, (Medtronic, USA) and Expressman GEC (APT, China). The size of most GEC is range from 5F to 8F. Pilot studies reported that 4F GEC facilitated stent delivery and thrombus aspiration [6, 7]. However, the relative increase in the backup support was lower in the 4-in-6 system than 5-in-6 system [8]. A 5-4F tapered GEC may balance the backup support and trackability between 4F and 5F GEC.

**Fig. 1:**
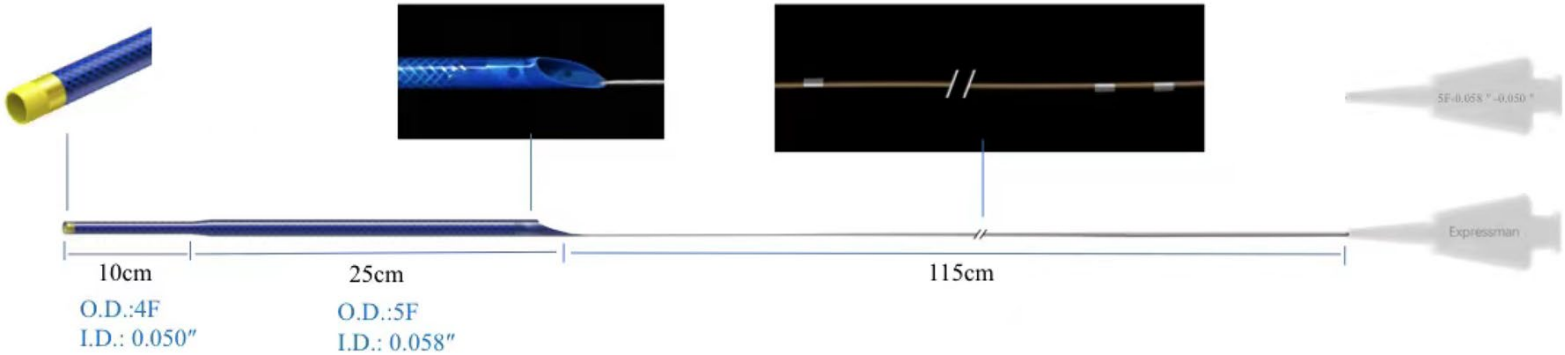
The 5-4F tapered Expressman guide extension catheter

Expressman GEC is a 150-cm catheter with similar outer diameter (1.70 mm) and inner diameter (1.42 mm) as compared to Guideliner V3 Catheter. The 35 cm rapid exchange segment and stainless steel braided layer design enable increased support and smooth delivery, which enable procedural success in the majority of type B2/C coronary lesion [5]. The Expressman™ 5-4F tapered GEC is a novel designed catheter used to provide additional backup support and access to distal lesions. The inner diameter is tapered from proximal (0.058″) to the distal tip (0.050″), which can accommodate for most stent delivery (Fig. 1).

In the study, we aim to evaluate the feasibility and safety with 5-4F tapered Expressman GEC used in complex lesion, including the depth of intubation, success rate of delivery and complication.

## Methods

### Study population

For the single-center, retrospectively cohort study, data were collected from patients underwent PCI between January 2021 and December 2022, at Zhongshan Hospital Fudan University. Either the 5F or 5-4F tapered Expressman was used to facilitate PCI procedure. The PCI procedure was performed by experienced interventional cardiologists. The Expressman GEC was used in the case of stent or balloon delivery failure, thrombus aspiration and active greeting technique in CTO. The success rate of device delivery was defined as the balloon or stent successfully crossing through the target lesion with Expressman catheter support. The success rate of procedure was defined as a successful stent implantation or drug-coated balloon (DCB) therapy in the targeted lesion area. The depth of intubation of the extension catheter and adverse outcome were recorded.

### Statistical analysis

Continuous normally distributed variables were expressed as mean ± SD, compared by one-way ANOVA and Student's t-test. Categorical variables were expressed as absolute numbers and percentages (%), compared using a Chi-square test or Fisher’s exact test. All statistical analysis was performed using IBM SPSS Statistics 25 software (IBM Corp., Armonk, NY). A value of *P* < 0.05 was considered as statistically significant.

## Results

In the study, there were 295 PCI procedures involving 5F Expressman device use, and 320 cases involving 5-4F tapered Expressman. A comparison of baseline clinical characteristics of these patients using 5F or 5-4F tapered GEC is presented in Table 1. The average age was 63.7 ± 11.0 years (63.6 ± 11.3 vs. 63.7 ± 10.8, *p* > 0.05) and 81.6% of the patients were male (84.1% vs. 79.4%, *p* > 0.05). 67.1% of population had hypertension (67.4% vs. 66.6%, *p* > 0.05), 38.3% of patients had diabetes (40.6% vs. 35.9%, *p* > 0.05), and 36.3% patients smoked (34.2% vs. 38.1%, *p* = 0.32). Prior revascularization by PCI (34.6% vs. 41.6%, *p* = 0.075) or CABG (2.4% vs. 3.8%, *p* = 0.32) was similar between the two groups.

**Table 1:** Patient demographics and clinical characteristics

	5F GEC	5-4F GEC	*p*
Age, years	63.6 ± 11.3	63.7 ± 10.8	0.84
Male	248 (84.1)	254 (79.4)	0.167
Diabetes mellitus	120 (40.6)	115 (35.9)	0.24
Hypertension	199 (67.4)	213 (66.6)	0.86
Hypercholesterolemia	76 (25.7)	92 (28.8)	0.39
Prior CABG	7 (2.4)	12 (3.8)	0.32
Prior PCI	102 (34.6)	133 (41.6)	0.075
Smoking	101 (34.2)	122 (38.1)	0.32

**Fig. 2:**
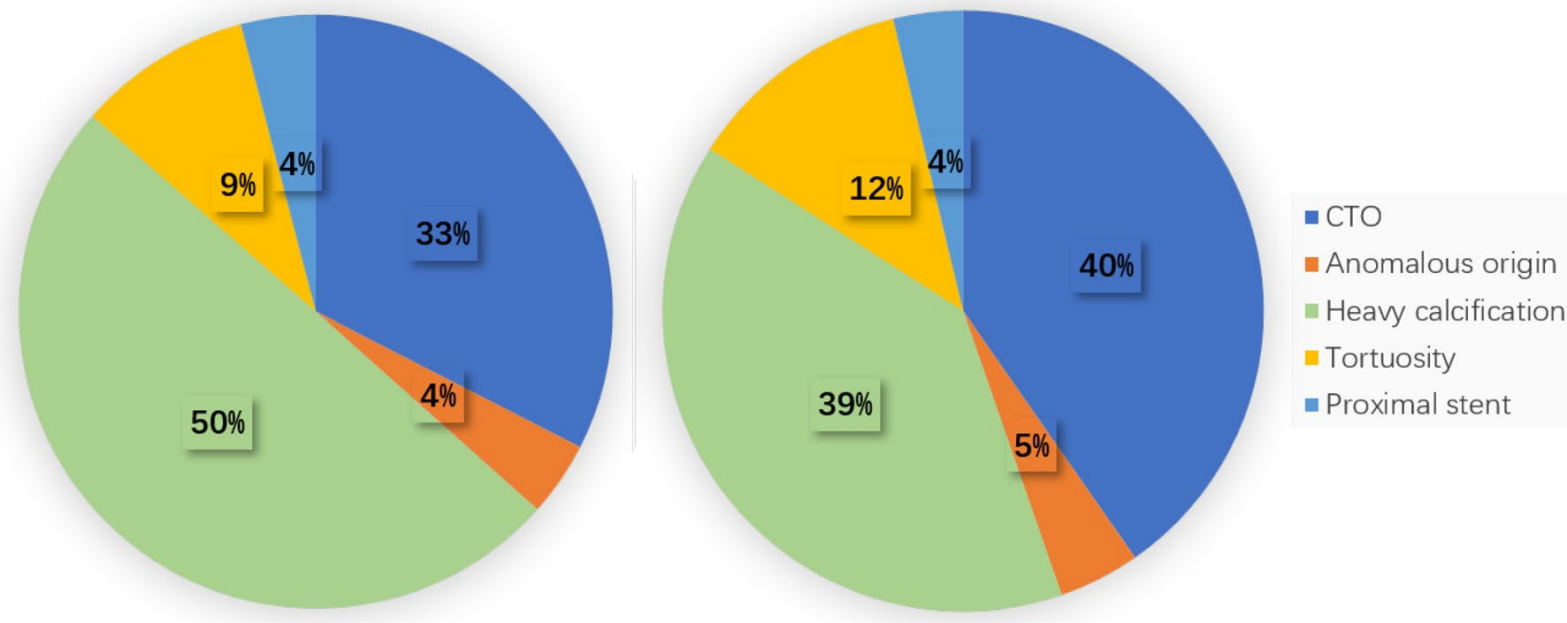
Indications for guide extension catheter use in PCI

Target vessels and lesion characteristics are shown in Table 2. The right coronary artery (RCA, 54.2% vs. 57.8%, *p* = 0.37) was the most common target vessel followed by the left anterior descending artery (LAD, 33.2% vs. 30.3%, *p* = 0.45) and left circumflex artery (LCX, 11.2% vs. 10.6%, *p* = 0.83). 5-4F GEC was frequently used in 36.6% of chronic total occlusion (32.5% vs. 40.3%, *p* < 0.05). 95% of cases were type C lesions (94.6% vs. 95.3%, *p* = 0.68). The  > 40 mm lesions accounted for 63.75% in 5-4F GEC group and 53.56% in 5F GEC group (*p* < 0.05). The most common indication for 5-4F tapered GEC use was CTO lesion, followed by heavy calcification, tortuosity, anomalous coronary artery origin and proximal stent (Fig. 2).

**Table 2:** Target vessels and lesion characteristics

	5F GEC	5-4F GEC	*p*
Target vessels			
Left anterior descending artery	98 (33.2)	97 (30.3)	0.45
Left circumflex artery	33 (11.2)	34 (10.6)	0.83
Right coronary artery	160 (54.2)	185 (57.8)	0.37
Vein graft	4 (1.4)	4 (1.3)	0.91
Target lesions			
Type B2/C lesion	16/279	15/305	0.68
Chronic total occlusion	96 (32.5)	129 (40.3)	0.046
Lesion length			
≤ 20 mm	32 (10.85)	31 (9.69)	
20–40 mm	105 (35.59)	85 (26.56)	
> 40 mm	158 (53.56)	204 (63.75)	0.031

Procedural data and complications associated with Expressman use are outlined in Table 3. Most procedures were performed via radial access. Femoral access was frequently used in 5-4F GEC group than 5F GEC group (6.4% vs. 11.6%, *p* = 0.027). Overall, the shapes of mother guiding catheter were extra backup left (EBU) in 263 cases, SAL in 220 cases, AL in 70 cases and Judkins in 62 cases, which were similar between two groups. Procedural involving rotational atherectomy accounted for 5% of cases (4.4% vs. 5.6%, *p* = 0.49). 5-4F tapered GEC was frequently used in active greeting technique (AGT) during CTO intervention procedure than 5F GEC (6.1% vs. 13.1%, *p* < 0.001). The average depth of intubation was 41.5 ± 19.6 mm for the 5-4F tapered GEC and 24.4 ± 15.1 mm for 5F GEC (*p* < 0.001). The rate of successful device delivery with 5-4F GEC was higher than 5F GEC (95.6% vs. 98.4%, *p* = 0.037). In 5-4F tapered GEC group, 21 cases (6.6%) were after failed mother–child technique of 5-in-6F. Pressure damping with 5F GEC occurred frequently than 5-4F GEC (7.4% vs. 2.5%, *p* < 0.05). Similarly, the incidence of intraoperative hypotension was higher in 5F GEC than 5-4F GEC (4.7% vs.1.9%, *p* < 0.05). Stent loss occurred in 2 cases with 5F GEC and 3 cases with 5-4F GEC (*p* > 0.05). The incidence of vessel dissection was 1.3%, occurring in 6 cases involving the 5F GEC, and 2 case involving the 5-4F GEC (*p* > 0.05). Ventricular tachycardia occurred in 2 cases with 5F GEC and 1 case with 5-4F GEC (*p* > 0.05). No death and Q-wave myocardial infarctions was observed in hospital.

**Table 3:** Procedural data of patients who underwent PCI using Expressman catheter

Variable	5F GEC	5-4F tapered GEC	*p*
Access			
Radial, no. (%)	276 (93.6)	283 (88.4)	0.027
Femoral, no. (%)	19 (6.4)	37 (11.6)	0.027
Shape of mother guiding catheter			
Extra backup (EBU)	129 (43.7)	134 (41.9)	0.64
Judkins	35 (11.9)	27 (8.4)	0.16
Short Amplatz left (SAL)	105 (35.6)	115 (35.9)	0.93
Amplatz left (AL)	26 (8.8)	44 (13.8)	0.054
AGT	18 (6.1)	42 (13.1)	0.003
Rotational atherectomy	13 (4.4)	18 (5.6)	0.49
Successful device delivery	282 (95.6)	315 (98.4)	0.037
Failed mother–child technique of 5-in-6F	–	21 (6.6)	–
Coronary dissection	6 (2.0)	2 (0.6)	0.12
Depth of intubation (mm)	24.5 ± 15.1	41.5 ± 19.6	0.001
Stent disruption or loss	2 (0.7)	3 (0.9)	0.72
Ventricular arrhythmia	2 (0.7)	1 (0.3)	0.52
Pressure damping	22 (7.5)	8 (2.5)	0.004
Intraoperative hypotension	14 (4.7)	6 (1.9)	0.045
All-cause death	0	0	

## Discussion

In the study, our data indicated that the 5-4F tapered Expressman GEC was superior to the 5F Expressman GEC to deeply intubate the coronary artery with less pressure damping. The two kinds of catheters both showed superior safety and deliverability in intervention of complex coronary lesion.

Expressman^™^ guiding extension catheter was designed as a rapid exchange catheter with 35-cm hydrophilic coating segment connected by a 115-cm nitinol shaft, which is currently available in size ranging from 5 to 6F. The device has proved safe and effective with the transradial interventions of complex coronary lesions. A novel designed Expressman^™^ GEC has a flexible tapered tip with gradually changed distal diameter. The inner diameter is tapered from proximal (0.058″) to the distal tip (0.050″), which can accommodate for most stent delivery. Compared to 5F Expressman catheter, 5-4F tapered catheter offer higher deliverability and deeper intubation for access to distal lesions. There was a significantly lower complication rate including pressure damping and intraoperative hypotension.

**Fig. 3:**
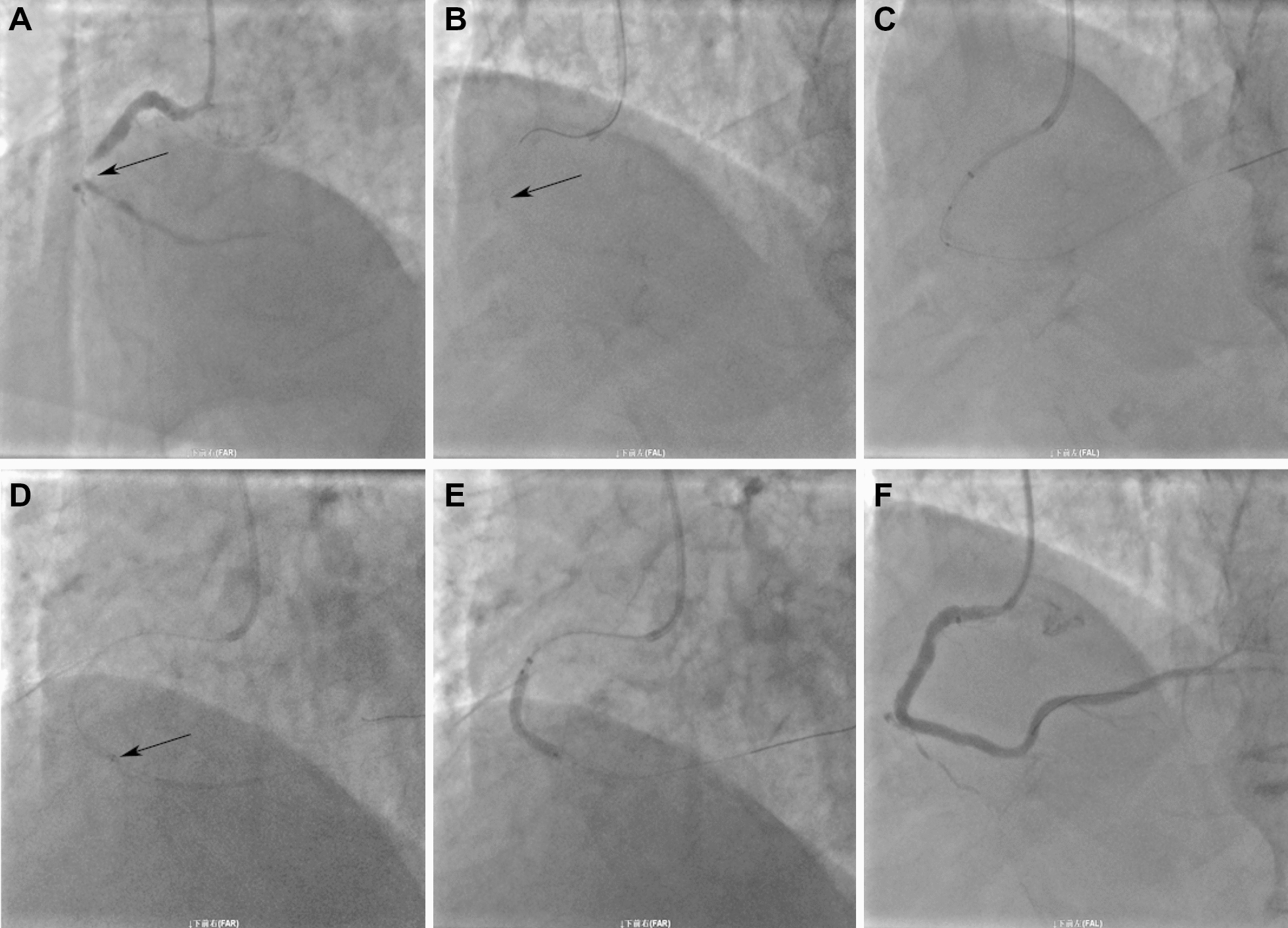
The use of 5-4F tapered Expressman GEC for severely calcified lesion. **A** Angiography indicated a subtotal occlusion in the right coronary artery (arrow). **B** X-ray showed calcified lesion with angulation. **C** With the support of 5F Guidezilla GEC, the lesion was dilated using a 2.0 mm and 2.5 mm balloon. However, the Resolute stent (3.0 × 22 mm) failed to cross the lesion. The 5F GEC was hampered at position of calcified lesion. **D** The 5-4F tapered GEC was advanced beyond the lesion with an anchor balloon technique. The stent was delivered beyond the target lesion via GEC. The arrow indicates the tip of the GEC. **E** The Resolute stents (3.0 × 22 mm and 3.5 × 22 mm) were positioned and deployed in the target lesion. **F** The final angiogram showed successful deployment of the 2 coronary stents with no visible arterial dissection

Previous data demonstrated that the backup support of catheter was associated with the diameters. The 7F guiding catheter provided additional backup support than 6F catheter [6, 8]. The backup support gradually increased when GEC was advanced beyond the tip of the mother guiding catheter. The relative increase in the backup support was lower in the 4-in-6 system than 5-in-6 system, which was inversely associated with the size of mother guiding catheter [8]. Many studies demonstrated the efficacy of a 4 Fr inner catheter for stent delivery [6, 7, 9]. Although the backup support of 4F child catheter was significantly lower, the trackability of the 4F GEC was superior to that of 5F GEC. In combination with the increased backup support and the superb trackability, 4F GEC achieved  > 90% success rate for lesions in which a balloon or stent failed to cross using conventional PCI techniques [6]. It is noteworthy that 5-4F tapered GEC may provide a unique characteristic that balance the backup support and trackability. Compared with 5F GEC, the 5-4F tapered GEC can be engaged deeply and provide higher success rate of device delivery. In the cohort, 6.6% cases were completed with 5-4F GEC after failed mother–child technique of 5-in-6F (Fig. 3). Furthermore, the 5-4F tapered Expressman GEC can inserted inside compatible guides over monorail support without the need to additional Y-connector, which brings convenience in PCI procedure. However, further study is required to compare the difference between a 4F and 5-4F tapered GEC in the backup force and the depth of intubation.

**Fig. 4:**
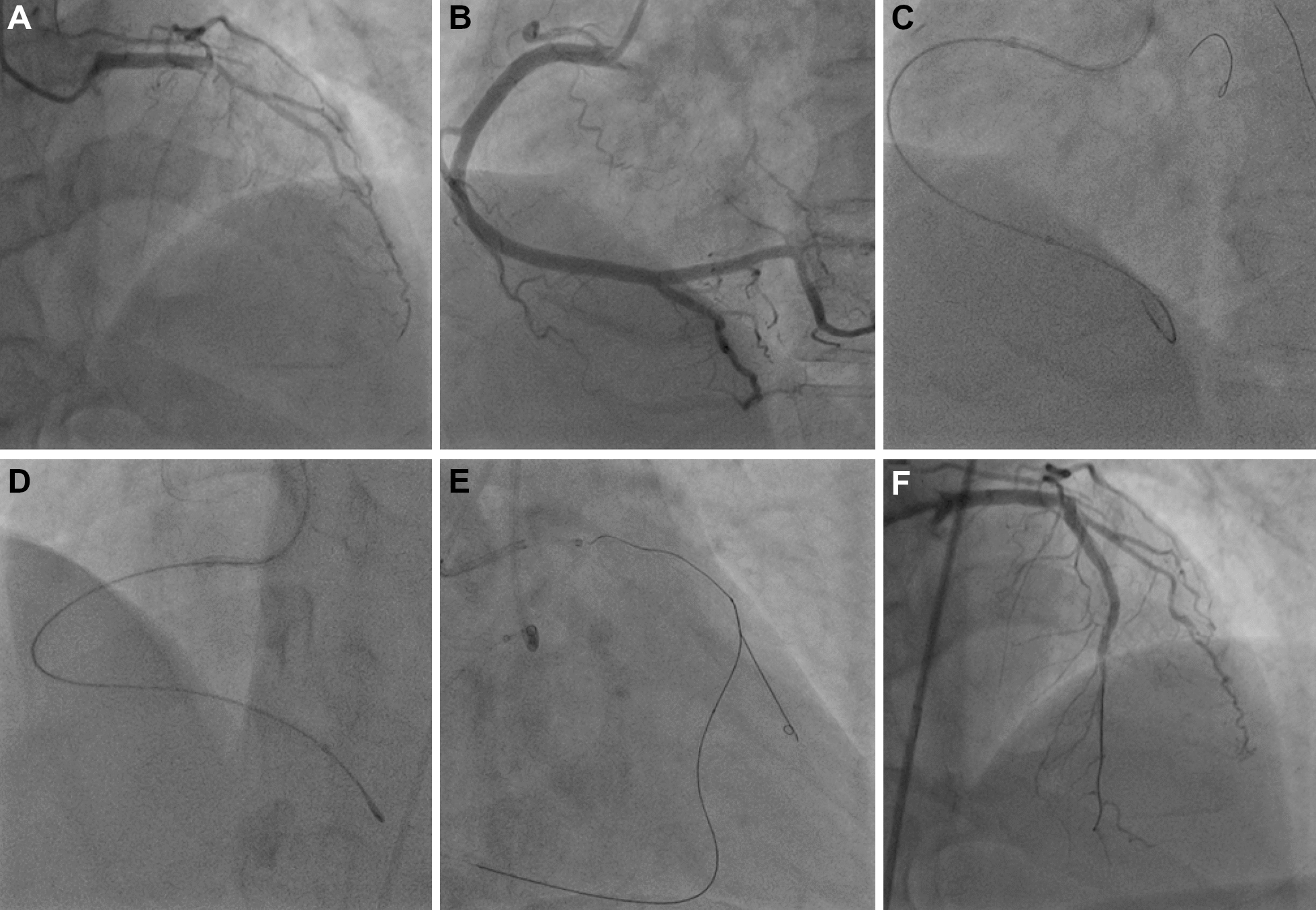
The use of 5-4F tapered Expressman GEC for chronic total occlusion. **A** Angiogram indicated a chronic total occlusion in the left anterior descending coronary artery (LAD). **B** Angiogram showed the right coronary artery. **C** and **D** After antegrade crossing failure, the Sion guidewire was advanced the septal perforating arteries with the support of microcatheter. However, the microcatheter failed to cross the septal branch. The 5-4F tapered GEC was used to increase backup support. **E** After the Sion guidewire and microcatheter was advanced into LAD, the Fielder XT-R was advanced the CTO lesion. The 5-4F tapered GEC was used for AGT. **F **The final angiogram showed successful deployment of the 2 coronary stents with no visible arterial dissection

GEC is of enormous help in controlled antegrade and retrograde tracking (CART) technique [10]. Active greeting technique is a feasible and safe technique that facilitates retrograde wire externalization with the use of GEC in CTO lesions [11]. It is necessary for AGT to advance GEC into proximal occlusion directly. The proximal lesions may limit the deep intubation of GEC. The 5-4F tapered GEC showed a superb trackability for AGT in CTO lesion due to the deeper intubation compared with 5F GEC. Thus, the 5-4F tapered GEC was more favored in AGT with 70.2% utilization in the study (Fig. 4).

Deep intubation of a 5F GEC into the coronary artery often causes pressure damping, especially in case of diffuse lesion. However, it should be noted that the 5-4F tapered GEC did not usually compromise the coronary flow with lower frequency of pressure damping. The small size profile of GEC may contribute to decrease the incidence of pressure damping. The incidence of intraoperative hypotension was also lower in 5-4F tapered GEC group than 5F GEC group. However, there were no differences between groups in incidence of coronary dissection, ventricular arrhythmia and all causes death.

The main limitation is that it is a single-center, retrospective cohort study, which might contain a potential selection bias. It is also noted that the experience and technique varied among operators. Additionally, the backup support of the 5-4F tapered GEC should be evaluated in in vitro experiments.

In conclusion, the 5-4F tapered GEC was superior to the 5F Expressman GEC in facilitating successful completion of PCI in the majority of patients with complex lesions. This novel tapered GEC may provide a viable alternative solution for the PCI procedure of complex coronary lesion.

## Data Availability

Original data are available on request to the corresponding author (liaojianquan@163.com or yao.kang@zs-hospital.sh.cn). The data are not publicly available due to privacy restrictions.
